# The Critical Functions of FGF2, LIF and IGF1 in the Improvement of In Vitro Embryo Production

**DOI:** 10.3390/biom16040487

**Published:** 2026-03-24

**Authors:** Paula M. Mangiavacchi, Kiho Lee, Bethany K. Redel

**Affiliations:** 1 Division of Animal Sciences, College of Agriculture Food and Natural Resources, University of Missouri, 920 East Campus Drive, Columbia, MO 65211, USA; pm9zz@missouri.edu (P.M.M.); kiholee@missouri.edu (K.L.); 2 National Swine Resource and Research Center, University of Missouri, Columbia, MO 65211, USA; 3 Plant Genetics Research Unit, Agricultural Research Service, United States Department of Agriculture, Columbia, MO 65211, USA

**Keywords:** in vitro environment, oocyte maturation, blastocyst, growth factors, signaling regulation, embryokines

## Abstract

In vitro embryo production (IVP) has emerged as a crucial tool in assisted reproduction and animal biotechnology. A key factor in this process is in vitro oocyte maturation (IVM), a critical process preceding fertilization that directly influences embryo quality. FLI supplementation, composed of fibroblast growth factor (FGF2), leukemia inhibitory factor (LIF), and insulin-like growth factor 1 (IGF1), has been shown to facilitate the IVM process to mimic essential aspects of in vivo oocyte development, and therefore, promote higher rates of oocyte maturation, embryonic viability, blastocyst formation, and improve the number of live animals born after embryo transfer. Individually or together, these components participate in signaling pathways that are crucial for improving oocyte competence and early embryo development. This review highlights the individual and combined roles of FGF2, LIF, and IGF1 in maturation and embryo culture medium, their influence on subsequent embryonic development, and their signaling pathways. Additionally, the incorporation of antioxidants and amino acids as supplementary components in combination with FLI is explored as a strategy to mitigate oxidative stress and enhance metabolic support during IVM and embryo culture. Together, these elements can significantly improve IVP outcomes, providing a potential pathway for optimizing the efficiency of embryo production in various species.

## 1. Introduction

In vitro embryo production (IVP) has been widely used to enhance the availability of elite genetics as a form of assisted reproductive biology. Its application, alongside other assisted reproductive technologies (ART), such as artificial insemination and embryo transfer, serves as an effective alternative for optimizing genetic improvement [1,2]. Beyond its role in genetic advancing, in vitro embryo culture also functions as a valuable model tool for studying and producing genetically modified animals [3,4,5]. Despite these advantages, the production of viable embryos remains challenging, primarily due to the fact that in vitro conditions used for oocyte maturation and embryo culture differ significantly from the in vivo environment across species.

Environmental and nutritional factors directly impact the fate of embryos [6,7]. Consequently, the success of IVP relies on critical factors that influence each stage, from in vitro oocyte maturation (IVM) to embryo development prior to implantation. Oocyte competency is particularly crucial, as it is a key stage preceding fertilization that directly affects embryo quality. Optimizing the conditions of IVM is therefore essential for improving oocyte competence and, consequently, increasing both the production and quality of embryos [8,9,10].

IVM media generally contain gonadotropins, such as luteinizing hormone (LH) and follicle stimulating hormone (FSH), along with follicular fluid (FF) and fetal bovine serum (FBS), to mimic in vivo environmental conditions. Although FBS has traditionally been used due to its rich content of growth factors and nutrients [11,12,13], it is often replaced with bovine serum albumin (BSA) due to batch-to-batch variability. This inconsistency is evident in the biphasic effects in FBS, in which undefined concentration of metabolites and growth factors can inhibit oocyte maturation and early embryonic cleavage, while promoting blastocyst development when present from the onset of compaction [14,15,16]. Similarly, the use of BSA can also have variable effects on cleavage and the development of morula and blastocyst stage embryos due to the significant variability between batches [17,18]. To reduce the variability often seen with using serum, the generation of serum-free medium has been investigated as a viable alternative. The use of polyvinyl alcohol (PVA), for example, in the oocyte maturation medium and embryo culture has shown positive results in the development and quality of the oocytes and embryos produced [19,20,21]. In addition, various supplements have been explored to enhance oocyte and embryo development [14,22]. For example, adding components such as amino acids, antioxidants, and growth factors to IVM and/or in in vitro culture (IVC) medium have demonstrated positive effects on oocyte maturation and embryo viability. A study conducted prior to Yuan et al. [4], in buffalo, demonstrated the potential of a conditioned medium obtained from the culture of umbilical cord embryonic cells for in vitro embryo culture. This medium, enriched with FLI, resulted in significant improvements in embryonic development rates [22]. The in vitro culture medium supplemented with 50% conditioned medium increased the cleavage rates to 70%, and the blastocyst rates to 24%. These findings underscore the critical role of these factors in embryonic development and highlight the potential of conditioned medium as a tool to address the limitations in the in vitro production of buffalo embryo oocyte maturation and embryo viability [8,22,23], in part by mitigating oxidative stress, promoting cell division, and supporting post-implantation placental development [9,23,24].

The FLI medium, comprising fibroblast growth factor 2 (FGF2), leukemia inhibitory factor (LIF), and insulin-like growth factor 1 (IGF1), was established with the primary goal of promoting oocyte maturation and subsequently supporting early embryonic development [4]. Each of these factors are well-recognized for their roles in cell development and differentiation, making them essential for creating an environment conducive to healthy embryo growth [8,25]. Individually, components of the FLI medium have distinct functions in supporting cell differentiation and survival while enhancing overall cell viability [26,27,28]. Together, these cytokines and growth factors provide the necessary support to improve oocyte competence and subsequent embryonic development, resulting in embryos with higher implantation potential and greater reproductive success [4,21]. More recently, the FLI medium has been combined with other additives to further enhance oocyte maturation rates and in vitro blastocyst formation [10,29,30,31], solidifying its value as a tool for assisted reproduction in multiple animal species.

The objective of this review is to highlight the critical functions of each component of the FLI medium and to explore how these factors synergistically contribute to improving in vitro embryo production through enhanced oocyte competence. This article encompasses the molecular mechanisms and signaling pathways associated with the FLI and illustrates how these cytokines and growth factors promote the success of in vitro embryo production and their potential applications across various animal models.

## 2. The Role of Cytokines and Growth Factors in Supporting In Vitro Oocyte Maturation and Embryonic Development

In vitro embryo production has made significant progress in recent decades, however, it remains associated with a high prevalence of pregnancy loss. In cattle, the quality of a transferred embryo is a crucial factor since the highest rate of loss occurs in the first weeks, during the peri-implantation period [32,33]. Considering the potential of this reproductive biotechnology, the development of an in vitro culture medium that supports embryonic development is critical. One promising approach has been the formulation of culture media with defined concentrations of molecules found in the reproductive tract—known as embryotrophic factors—that can enhance oocyte quality and subsequently embryo development [33,34,35]. However, many of these factors, such as cytokines, hormones, and growth factors, are often present in fetal serum, where their concentrations are poorly defined and vary among different batches [36], underscoring the need for more controlled and standardized culture systems.

Embryotrophic factors identify cellular receptors capable of modulating gene expression through activation pathways, thereby influencing critical cellular functions such as metabolism and molecule secretion [37]. Cytokines and growth factors act in autocrine, endocrine, and paracrine manners to support all stages of conceptus survival and progeny health, thus playing a crucial role in communication between the female reproductive tract and developing embryo(s) [38]. In contrast, embryotoxic factors such as the pro-inflammatory tumor necrosis factor and interferon gamma suppress embryonic development [39]. The ovarian cycle, nutritional or hormonal factors can impact the balance of cytokines, therefore affecting the activity of embryotrophic and embryotoxic factors [38,40]. This balance under the in vitro microenvironment is influenced by the composition of media, which may induce cellular stress and epigenetic changes during fetal development [41].

Cytokines such as interleukins, interferons, tumor necrosis factors, and chemokines play essential roles in cellular communication, hormone regulation, and the immune system, cellular apoptosis, and embryonic attachment or implantation. In humans, high concentrations of interleukins 1-β and interleukina-8 are associated with increased implantation and pregnancy rates [42,43]. The interleukina-6 (IL-6) family, which includes LIF, is produced in the oviduct and endometrium during bovine pregnancy [33,44].

Colony stimulating factors (CSFs) act through the activation of phosphatidylinositol 3-kinase (PI3K)/protein kinase B (AKT)/extracellular signal-regulated kinases (ERK) pathways during reproductive events that are crucial for trophoblastic invasion and cellular migration [45]. Specifically, granulocyte colony stimulation factor (G-CSF) increases cellular differentiation and adhesion, trophoblastic invasion, and placentation development [45,46]. In humans, subcutaneous G-CSF administration in couples with recurrent implantation failure (RIF) has demonstrated lower miscarriage rates and higher live birth rates [47]. Additionally, granulocyte-macrophage colony stimulating factor (GM-CSF), naturally present in ovarian follicles, has been shown to enhance cell proliferation and embryo production when supplemented in in vitro cultures. This effect has been observed in mice [48], cattle [49], humans [50], and pigs [51]. In bovine, FLI supplementation combined with transforming growth factor beta (TGF-β) and GM-CSF significantly improved blastocyst quality by increasing the number of cells in both the inner cell mass (ICM) and trophectoderm (TE) [52]. This improvement was observed only when these factors were used together, as their individual use did not result in a beneficial effect.

Growth factors are spatiotemporally present during the preimplantation period of embryo development [23,53]. In pigs, supplementation with epidermal growth factor (EGF) to IVM promotes cumulus cell proliferation and reduces apoptosis by maintaining gap junctions [54]. Similarly, adding amphiregulin (AREG), an EGF-like factor, during pig IVM enhances cumulus cell expansion and improves the competence of cloned embryos derived from these oocytes [55].

The use of these factors in the in vitro environment has become a promising strategy for improving embryonic development. The combination of FGF2, LIF, and IGF1 emerges as a consistent alternative for optimizing oocyte maturation and embryonic development across various species.

### 2.1. FGF2 (Fibroblast Growth Factor 2)

Fibroblast growth factors are a family of 22 polypeptides conserved in vertebrates that regulate cell proliferation, migration, and differentiation, playing key roles in tissue repair [56,57]. They act through four tyrosine kinase receptors (FGFR1–4) and are classified into seven subfamilies, according to their actions. The FGF1 subfamily, consisting of FGF1 and FGF2 (also called ‘basic fibroblast growth factor’—bFGF2) was first identified in the 1970s and functions via paracrine signaling, secreted into the extracellular environment and binds tyrosine kinase receptors, FGFRs [57].

FGF proteins promote cell proliferation and angiogenesis by stimulating factors such as vascular endothelial growth factor (VEGF), G-CSF, EGF, and GM-CSF [57,58,59]. In myoblasts, FGF2-induced cell proliferation occurs through the activation of signaling cascades downstream of its FGF receptor (FGFR), including the sarcoma family kinases (SRC), mitogen-activated protein kinase (MAPK), and extracellular signal-regulated kinase (ERK) pathways [60,61]. In addition, FGF2 signaling, along with the phosphatidylinositol 3-kinase (PI3K) pathway and ERK activity, have been implicated in regulating cellular senescence and apoptosis [61] in fibroblasts [62].

Supplementing FGF2 to in vitro embryo culture environments has produced varied results in different species, such as cattle [15], pigs [63], sheep [64], mice [65], and chickens [66]. These differences reflect variations in biological responses and cell signaling mechanisms of FGF2-activated pathways across species, as well as differences in the FGF2 source, medium composition, and the environment of the in vitro culture conditions [24,28,67].

FGFs are known to positively influence trophectoderm development in mice through the fgf-ERK signaling pathway. The formation of trophectoderm cells is essential for the development of the extraembryonic ectoderm, and defects in their formation and function can lead to failures in early embryonic development [65]. For example, mouse embryos lacking functional fgf4 or fgfr2 resulted in embryo lethality during the pre-implantation period due to failures in trophectoderm cell expansion [65,68]. Additionally, in mice, fgf2/fgfr2 promotes oocyte maturation, and the use of specific inhibitors, such as SU402, can reduce the production of 8-cell embryos and blastocysts [69].

FGF helps embryonic stem cells (ESCs) maintain their pluripotent state [63,70]. The presence of FGF2 in cultured embryonic germ cells from fetal pig gonads supported pluripotency and differentiation [71], and also induced the proliferation and differentiation of ESCs derived from the ICM [63]. However, FGF2 alone was insufficient to maintain ESC cultures requiring the combination with other components such as activin A and the WNT signaling activator CHIR99021 [63]. Similar findings were reported in another study where FGF2 combined with interleukins, oncostatin M, EGF, and LIF supported establishing in vitro ESC cultures from pig ICM [70].

In the reproductive context, FGF is present in the ovaries and their components, including follicular fluid, granulosa cells, oocytes, and cumulus cells [72]. The concentration of FGF in follicular fluid varies according to follicle size and oocyte maturity. This has been described in humans (basic FGF—bFGF; [73]), buffalo (FGF2; [72,74]), cattle (FGF2; [75]), and pigs (FGF2; [76]). In buffalo, 5–20 ng/mL of FGF2 significantly increased the cumulus oocyte complex (COC) diameter, oocyte maturation, and blastocyst total cell number [28,77]. In cattle, FGF2 is expressed in theca and cumulus cells, where it regulates follicle recruitment, cumulus expansion, and oocyte meiosis, partly through prostaglandin-endoperoxide synthase 2 (*PGTS2*) and hyaluronan synthase 2 (*HAS2*) expression [10,15,24,69]. It also prevents granulosa cell apoptosis and is upregulated by FSH and LH during IVM [69,78] and increases the number of oocytes reaching germinal vesicle breakdown after 6 h of maturation [24]. Lastly, a combination of embryotrophic factors—EGF, IGF1 and FGF2—promoted trophoblast proliferation, blastocyst expansion [79] and increased the post-transfer survival rate more transferable embryos after 7 days of in vitro culture [25].

Supplementation with low molecular weight human FGF2 has been shown to enhance bovine oocyte maturation, blastocyst production, and cell proliferation [80,81]. However, other studies have reported no detectable effects of FGF2 on maturation or cleavage, likely due to differences in media composition or timing of supplementation [82,83]. These discrepancies may stem from differences in the media used and/or the specific stages of in vitro culture during which FGF2 supplementation was applied, which can significantly influence the responsiveness of the cells to this growth factor.

Studies confirm the production of FGF2 in the uterus of various domestic animals, including pigs [84], mice [85], rabbits [86], and cattle [87], showing the FGF2 potential to induce the expression of synchrony factors. A previous study in sheep added either 100 or 500 ng/mL of FGF2 to in vitro embryo culture, and the supplementation did not affect blastocyst production or total cell number. However, FGF2 supplementation did alter gene expression in ICM cells, where the epiblast marker, *NANOG*, was downregulated and the hypoblast marker, *GATA4*, was upregulated [88].

Collectively, these findings indicate that FGF2 contributes to the regulation of oocyte developmental competence in in vitro embryo production systems and promotes early embryonic development. However, different studies suggest that these positive effects may be more pronounced when FGF2 is combined with other growth factors and cytokines such as IGF1, LIF, EGF, or TGF-β [25,52,89].

### 2.2. LIF (Leukemia Inhibitory Factor)

The leukemia inhibitory factor, a glycoprotein belonging to the IL-6 interleukin family, was discovered through studies on myeloid leukemia cell lines [90,91]. Members of the IL-6 family are grouped based on their structure and receptor specificity, although they all share a common cellular receptor, known as gp130 [92]. The secondary receptor capable of binding to the gp130 differentiates the members of this family as the LIF receptor for the cytokine LIF [91]. The gp130 receptor is expressed in all cell types in the body, whereas the secondary receptors are present in more cellular specific patterns, thus determining the specific action of each cytokine [91,93]. Multiple LIF receptors are present in human ESCs, as well as in various tissues such as the liver, bone, uterus, kidneys, and cells of the central nervous system [90,94,95], highlighting their role in development, reproduction, bone remodeling [95], and in the neuromuscular and hematopoietic systems [90,96].

In mice and human ESCs, LIF plays a role in maintaining the pluripotent state of these cells by stimulating their self-renewal. Its receptors are found in the blastocyst, endometrium, and trophoblast, suggesting a coordinated action between maternal and embryonic tissues [94,97]. In both species, LIF is highly expressed in the endometrium, playing a key role during embryo adhesion and implantation [98,99].

In human and mouse oocytes, the addition 1000 of ng/mL LIF during in vitro maturation stimulates cumulus cell expansion [100]. In combination with 0.2 IU/mL FSH, the improvements were even statistically significant in the mouse oocyte competence, as reflected by the rate of 2-cell embryo formation. Approximately 80% of these embryos developed into blastocysts, and 76% resulted in live births compared to 56% and 59% in the groups treated with FSH or LIF alone, respectively. In contrast, adding 100 ng/mL human or mouse LIF during in vitro bovine embryo culture reduced blastocyst formation and inner cell mass, respectively [101].

LIF receptors can also be found in cumulus cells and oocytes in pig [26] and sheep [102]. In bovine, for example, the LIF receptor subunit alpha (LIFR) was also detected in both, whereas the IL-6 receptor (IL6R) was identified only in cumulus cells [102], reflecting the difference in the effects observed when LIF and IL-6 were supplemented during in vitro maturation. A total of 25 ng/mL of LIF promoted higher *AREG* transcription along with a trend toward increased *HAS2* transcription after 22 h, while supplementation with 25 ng/mL of IL-6 led to a reduction at the same time point, which may be associated with the lower rate of mature oocytes obtained when IL-6 was added to maturation. While LIF did not improve the nuclear maturation of oocytes, it increased the development to blastocyst stage compared to the control, revealing a potential improvement to bovine oocyte competency.

The addition of 25 ng/mL of LIF to IVM medium also increased bovine oocyte maturation and mitochondrial potential [103]. Moreover, it improved embryo development after somatic cell nuclear transfer (SCNT) by increasing the total number of cells and reducing embryonic apoptosis, which resulted in a significantly higher pregnancy rate in the LIF-treated blastocysts (18%) compared to the control blastocysts (6%) on days 110–120 (*p* < 0.05). Different studies have shown that embryos produced by SCNT are less efficient due to failures in epigenetic reprogramming, leading to high embryonic mortality and/or development of Large Offspring Syndrome (LOS) [104,105,106].

Finally, 25 ng/mL of LIF improved cleavage, blastocyst formation, and cell number [107] when included during maturation prior to vitrification of bovine oocytes [108]. LIF treatment reduced DNA fragmentation and restored blastocyst rates to levels comparable with non-vitrified oocytes. Molecularly, LIF maintained normal gene expression profiles in vitrified oocytes, supporting its role in preserving developmental competence [108]. In contrast, the same research group reported no significant improvement in cleavage or blastocyst percentages following LIF supplementation during bovine oocyte maturation [109]. However, LIF did modulate the expression of several genes, such as developmental pluripotency associated 3, nucleophosmin/mucleoplasmin 2, and zygote arrest 1 in 2- and/or 8-cell embryos. Both are maternal-related genes associated with the regulation of DNA methylation, transcriptional control, and nuclear organization, respectively [110,111,112].

These findings suggest that under standard IVM conditions, LIF primarily influences molecular pathways without necessarily translating into measurable developmental gains. Its beneficial impact became particularly evident under the stress conditions imposed by vitrification, suggesting that LIF may function as a protective or competence-enhancing factor whose effects are more pronounced when oocytes are subjected to cryoinjury.

The supplementation of 1000 IU/mL of LIF to pig IVM increased the percentage of oocytes developing to metaphase II (MII) [26]. Additionally, there was increased expression of LIF and its receptors in oocytes and early cleavage stages (2- and 4-cell embryos), though blastocyst formation was reduced. A similar pattern of improvement in porcine MII oocyte rates was observed in the study by Wasielak et al. [113], although the addition of 1000 IU/mL LIF from Days 5 to 8 during in vitro bovine embryo culture resulted in significantly lower percentages of blastocysts, total cell number, and inner cell mass [114], highlighting the importance of dose and timing in LIF supplementation.

In pigs, embryo transfer has a limited efficiency, in part due to the higher embryo mortality rate during the pre- and peri-attachment periods (>50%), or the lack of maternal immunological tolerance [115,116]. LIF plays a role in maternal immunological modulation to promote embryo acceptance by regulating embryo–endometrial interactions and uterine receptivity [99,116]. LIF-mediated immunomodulation occurs through the induction of an anti-inflammatory response in the uterus, involving macrophage recruitment to the endometrium via activation of the STAT3 signaling pathway [117]. In addition, LIF increases the expression of the anti-inflammatory cytokine IL-10 and promotes the recruitment of regulatory T cells, which are responsible for maintaining immunological homeostasis [118]. The expression of LIF and LIFR were significantly lower in the pig endometrium during attachment after embryo transfer compared to artificial insemination [116], indicating that altered LIF expression may disrupt this immunological modulation, contributing to the higher embryonic mortality observed following embryo transfer procedures.

LIF also promotes follicular growth by stimulating the transcription of gap junction-related genes in sheep [119,120]. Sheep cumulus–oocyte complexes recovered from large follicles (550 µm) showed higher proportions of MII oocytes when matured with LIF and FSH (56%) compared with LIF alone (38%). In addition, LIF independent of FSH stimulated the expression of B-cell lymphoma 2 (*BCL2*), *BCL2*-ssociated X, apoptosis regulator (*BAX*), and connexin-43 and connexin-37, genes associated with apoptosis regulation and gap junctions, respectively [120].

In yak oocytes, supplementation with 50 ng/mL of LIF during IVM improved oocyte maturation rates, mitochondrial activity, actin integrity, and blastocyst cell number [121]. This improvement was supported by lower levels of reactive oxygen species, and the increase in *BCL2* and survivin, besides a decrease in the expression of caspase-3.

The beneficial effects of LIF are also observed when combined with other molecules. In guinea pigs, supplementation with 1000 IU/mL of LIF combined with 100 µM cysteamine (Cys) in the IVM medium resulted in a significant increase in MII oocyte percentages [122]. Furthermore, treatment with LIF and Cys positively influenced oocyte quality: type I oocytes (with four or more cumulus cell layers) showed significantly higher MII rates (61.8%) compared to type II oocytes (with one to three cumulus layers), which showed only 29.5% MII. The use of Cys in IVM media has previously been reported in pigs [123], cattle [124], and goats [125] and is often associated with reduced oxidative stress by increasing intracellular glutathione (GSH) levels in oocytes.

Despite the promising results reported with LIF supplementation during in vitro oocyte maturation across different species, some studies have shown inconsistent or contradictory outcomes, with no clear improvement in embryo development [36,101,114]. Variations in LIF concentration, culture conditions, and media composition are likely to contribute to these discrepancies. Notably, several of these studies were conducted under earlier in vitro systems, which have since undergone substantial optimization in terms of IVM protocols, culture media formulation, and associated biotechnological procedures. Therefore, the effects of LIF may become more consistent when optimal concentrations are established and applied in combination with other embryokines within chemically defined media systems.

### 2.3. IGF1 (Insulin-like Growth Factor 1)

IGF is an insulin-like growth factor directly associated with growth hormone (GH). Discovered in the 1950s, IGF was initially linked to the biosynthesis of muscle, adipose, and brain tissue [126,127]. According to the somatomedin hypothesis, GH does not act directly on target tissues to promote growth, instead it induces the production of secondary mediators [128]. In rats, GH stimulated the production of a molecule that acted on cartilage and liver tissues, leading to the conclusion that GH alone was not sufficient to promote growth. This molecule, called somatomedin (types A, B, and C), not only stimulated cell proliferation and growth but also exhibited insulin-like activity in the liver. Structural identification of IGF1 and IGF2 in human serum in the late 1970s clarified that somatomedin C corresponds to IGF1 and somatomedin A to IGF2 [129,130].

IGF1 and IGF2 both support cell growth and proliferation; however, IGF2 is an imprinted gene with a crucial role following in vitro fertilization, acting directly to ensure successful embryonic development [106,131]. Alterations in IGF2 expression patterns are commonly linked to the development of embryonic disorders and syndromes caused by the in vitro environment [105,106].

The circulation of IGF 1 and 2 occurs through binding to a family of six proteins called insulin-like growth factor binding proteins (IGFBPs), which transport them to target tissues and may also have IGF-independent effects [132]. In mice, the triple knockout of IGFBP3, 4, and 5 induced a reduction of up to 20% in adult size, which was also associated with low levels of IGF1 [133]. IGFBP proteases have also been detected in the serum of pregnant women, where they can degrade IGFBPs and release IGF, thereby facilitating the binding of insulin-like growth factors to their specific receptors [134].

Pregnancy-associated plasma protein A (PAPP-A), an IGFBP protease identified in the placenta, is capable of cleaving IGF from IGFBP, allowing it to bind to its cellular receptor [135]. In mice, PAPP-A knockout leads to reduced growth [136], and in humans, mutations in the PAPP-A2 gene have also been linked to IGF deficiency and short stature [137]. Commonly secreted by granulosa cells, PAPP-A is directly associated with fertility parameters, such as the number of COCs produced and the levels of progesterone and estradiol during ovulation, and is thus considered a marker of follicular selection [138,139].

In cattle, the addition of PAPP-A and IGF1 to the maturation medium did not affect oocyte competence, gene expression, or mitochondrial membrane potential [139]; although others have reported that PAPP-A and IGF1 are able of modulating lipid metabolism and mitochondrial potential [140]. PAPP-A and IGF1 are also capable of reducing reactive oxygen species (ROS) levels, regulate genes related to embryonic quality, and produce higher number of blastocysts [139]. This suggests that PAPP-A have dual action as an IGF1 releaser and as an adjuvant to the antioxidant effects of IGF1 during in vitro embryo production.

The cellular action of IGF1 occurs through the transmembrane receptors, insulin receptor and insulin-like growth factor 1 receptor (IGF1R), with IGF1R also mediating IGF2 binding to the cell [126]. IGF1 then activates different intracellular signaling cascades, inducing anti-apoptotic and cell survival responses [27], and can be found in various cell types, including the oocyte [141,142]. Failures in the transcription of this receptor have been associated with defects in organ growth and development [126]. *IGF1R* mRNA was also detected in oocytes and embryos at all developmental stages after IGF2 supplementation in bovine in vitro maturation medium [143], indicating its participation in cumulus cell expansion and nuclear maturation of bovine oocytes [54]. The action of IGF1 was more evident in conjunction with EGF in the maturation medium, resulting in a higher proportion of MII oocytes [144]. EGF, in combination with IGF, act on granulosa cells as an adjuvant to FSH and their presence in the uterus is associated with the induction of cell proliferation [145].

IGF1 treatment also contributes to the development of primordial follicles in sheep [146], bovine [15], and humans [147], as well as to the reduction in follicular atresia [148]. In the in vitro culture of follicular tissue, treatment with 100 ng/mL IGF1 resulted in lower DNA fragmentation rates and greater follicular activation and growth after 7 days of culture [146]. However, the dose of IGF1 used can exert differential effects on follicular development depending on the developmental stage of the follicle [149]. For example, 20 ng/mL IGF1 has been shown to increase the diameter of preantral bovine follicles [150], indicating a stimulatory role at early stages of folliculogenesis while the exposure to 1000 ng/mL negatively affected follicular morphology and granulosa cell layer integrity in these same preantral follicles [151]. Interestingly, the same high concentration (1000 ng/mL) demonstrated beneficial effects in small-sized follicles (165–215 μm), but not in medium- and large-sized follicles, suggesting a stage-dependent response to IGF1 stimulation [152]. These findings show that IGF1 action is highly context-specific, and that supraphysiological concentrations may either be supportive or detrimental, depending on follicular maturity and the reproductive environment.

IGF1 is also able to mitigate the adverse effects of heat shock, demonstrating thermoprotective action by reducing the damage caused by high temperatures in the in vitro environment [153,154]. Heat stress (HS) is a physiological condition that occurs in response to several environmental factors, such as temperature, humidity, and atmospheric pressure, which can increase ROS, directly affecting sperm production [155,156]. Supplementation of IGF1 in mouse and sheep in vitro maturation medium mitigated the adverse effects of heat shock [153,154] through the regulation of genes associated with stress and apoptosis. Under IGF1 action, cells are able to activate antioxidant regulation genes such as superoxide dismutase 1 and 2 and glutathione-disulfide reductase, in response to oxidative stress [157]. The induction of elevated temperature (41 °C) during IVM of bovine oocytes revealed that lower concentrations of IGF1, such as 12.5 ng/mL and 25 ng/mL, are effective in mitigating the negative effects caused by heat stress [153]. Both concentrations reduced DNA fragmentation and, maturation with 12.5 ng/mL, observed a significant increase in the blastocyst rate on Day 8 (30%) compared to the untreated oocytes (15%) and to the concentration of 100 ng/mL (16%). Similar results were reported by Rodrigues et al. [154], who observed a thermoprotective effect with 25 ng/mL of IGF1, reflected in higher blastocyst rates.

The combined supplementation of 100 ng/mL IGF1, 50 µM coenzyme Q10 (CoQ10), and 1 µM melatonin was also able to reverse the effects of HS (41 °C) induced at the time of bovine in vitro fertilization. This treatment reduced ROS levels and apoptosis, in addition to increasing the mitochondrial membrane potential and upregulating genes related to ATP transport, cell adhesion, and maternal recognition of pregnancy [158].

In sheep, the combined supplementation of 100 ng/mL IGF1, 50 ng/mL EGF, and 25 µg/mL Connexin 37 (CX37) during IVM resulted in a significant improvement in nuclear maturation after oocyte vitrification [159]. EGF, IGF1, and CX37 supplementation also favored post-thaw viability in bovine oocytes, reflected in higher blastocyst rates, elevated GSH levels, and increased mitochondrial activity [9]. The positive effect on embryonic development was even more evident when these three components were used together with FSH and LH, resulting in higher cleavage (90% vs. 81%) and blastocyst (50% vs. 40%) rates, in addition to higher levels of ATP, GSH, and transzonal projections (TZPs) and reduced ROS levels.

In summary, the addition of IGF1, alone or in combination with other factors during in vitro maturation, enhances oocyte maturation, post-thaw viability, embryonic developmental competence, increases mitochondrial activity, and reduces oxidative stress. These findings underscore the synergistic potential of combining IGF1 with other growth factors and signaling molecules in defined culture media to optimize oocyte quality and embryo production, particularly under challenging environmental or experimental conditions. However, the effectiveness of IGF1 supplementation appears to be dose-dependent and context-specific. Optimal concentrations may differ depending on the reproductive objective, such as superovulation protocols versus in vitro embryo production systems, and should be adjusted accordingly. Moreover, physiological IGF1 levels vary among species, suggesting that species-specific adjustment is essential to maximize biological efficacy and avoid potential adverse effects associated with supraphysiological exposure.

## 3. Signal Transduction Pathways Activated by FGF2, LIF, and IGF1 in Mammalian Cumulus Cells and Oocytes

In mammals, the three components of the FLI medium work synergistically in cumulus cells and oocytes to enhance in vitro oocyte competence and, consequently, embryonic development. By coordinating multiple cellular processes, a complex network of signal transduction pathways activates diverse signaling cascades and modulates gene expression. These activated pathways create more favorable conditions for embryos to progress through the various stages of development.

**Figure 1:**
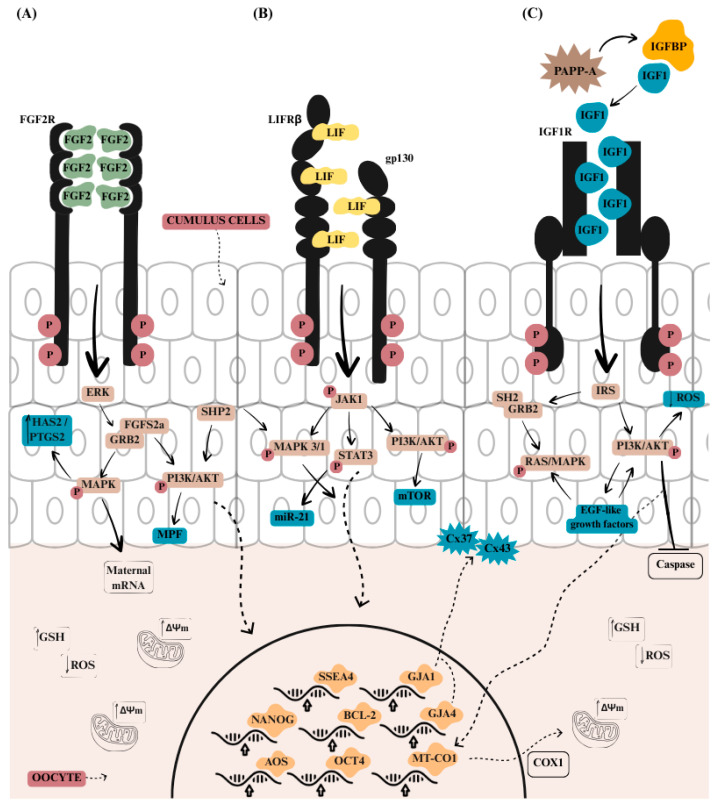
Signaling pathways activated by fibroblast growth factor 2 (FGF2), leukemia inhibitory factor (LIF) and insulin-like growth factor 1 (IGF1) in cumulus cells and oocytes in mammals. The receptors are illustrated in cumulus cells for simplicity, although they are also expressed in oocytes. (**A**) FGF2 binds to FGFR2, activating the ERK/MAPK and PI3K/AKT pathways in cumulus cells, promoting HAS2/PTGS2 expression and maternal mRNA regulation. (**B**) LIF interacts with LIFRβ and gp130, triggering JAK1/STAT3, MAPK, and PI3K/AKT/mTOR signaling, leading to miR-21 expression, cumulus–oocyte communication (Cx37/Cx43), and regulation of pluripotency- and survival-related genes. (**C**) IGF1 activates IGF1R through the IRS/PI3K/AKT and RAS/MAPK cascades, modulated by IGFBPs and PAPP-A, enhancing EGF-like growth factor signaling, mitochondrial activity, reducing apoptosis, and improving developmental competence. Solid black arrows indicate direct signaling pathways activated by growth factors and cytokines. Upward and downward solid black arrows within the boxes represent upregulation and downregulation, respectively, of glutathione (GSH), reactive oxygen species (ROS), and/or mitochondrial membrane potential (ΔΨm). Dashed arrows represent indirect effects or putative regulatory mechanisms linking signaling pathways to downstream responses. Open arrows within the nucleus indicate transcriptional regulation and changes in gene expression. All information highlighted here was obtained from different species, such as pigs [26], sheep [147], cattle [161], and humans [162]. The schematic illustration was created using Canva software 2025 (Canva Pty Ltd., Sydney, Australia).

FGF2, LIF and IGF1 act in the cumulus cells and the oocytes through different pathways (Figure 1). FGF2 activates the MAPK signaling pathway, which promotes cell proliferation and differentiation [58], while LIF helps maintain pluripotency by activating the Janus kinase (JAK)/Signal Transducer and Activator of Transcription 3 (STAT3) pathway, preventing premature differentiation [160]. Meanwhile, IGF1 boosts cell growth and metabolism through the PI3K/AKT signaling pathway, ensuring the energy and nutrient supply for the developing embryo [126].

Upon contact with FGFR receptors, FGF2 induces a structural change in their domains, causing the dimerization of different signaling pathways that are activated individually or through co-stimulation [58] (Figure 1A). Receptor phosphorylation leads to the binding of fibroblast growth factor substrate 2α (FRS2α), which recruits growth factor receptor bound protein 2 (GRB2), stimulating the activation of the RAS/RAR/MAPK signaling pathway (ERK, p38, JNK) and the PI3K/AKT pathway [58,163]. FGF2 acts on cumulus cell expansion by activating the c-Mos/MAPK pathway, which induces maturation promoting factor (MPF), essential for meiosis resumption, increased polar body extrusion, and meiotic spindle formation [69]. The increase in the c-Mos/MAPK pathway through the joint action of FGF2, LIF, and IGF1 is associated with increased nuclear maturation in bovine oocytes supplemented with FLI [164].

The protein kinase ERK belongs to the MAPK family, which plays a role in the transduction of extracellular signals to the nucleus, where it activates several transcription factors, such as c-MYC, the AP-1 complex, and ELK1. These transcription factors are responsible for regulating the gene expression of pluripotency genes, tissue remodeling, and angiogenesis, responsible for cell proliferation, differentiation, and survival [165]. In cattle, the phosphorylation of ERK1/2 via FGF2 has already been reported as one of the mechanisms responsible for triggering the resumption of meiosis [161,166]. The accumulation of ERK after the addition of FGF2 to the in vitro culture medium of pluripotent embryonic cells increased in a time-dependent manner, suggesting that this pathway plays a major role in maintaining cellular pluripotency [71].

LIF primarily activates the JAK1 kinase pathway, initiating a phosphorylation cascade that stimulates the STAT3, MAPK, and PI3K pathways [90] (Figure 1B). JAK1 then acts as a recruiter of STAT3, which in turn regulates the transcription of genes involved in anti-apoptosis, cell cycle progression, lipid metabolism, and differentiation, thereby preventing premature differentiation [94,109,120,160].

In pigs, the levels of phosphorylated STAT3 protein progressively increased in cumulus cells after LIF supplementation, disappearing after 44 h of maturation, while the total STAT3 levels remained constant [26]. Supplementation with 25 ng/mL of LIF during the in vitro maturation of bovine oocytes increased the phosphorylation of MAPK3/1 and STAT3 (*p* < 0.05) [107]. On the other hand, the inhibition of both pathways reduced the MII rate; MAPK inhibition allowed the partial recovery of MII oocytes with LIF, while JAK inhibition completely abolished LIF’s positive effects, resulting in a marked reduction in the MII percentages. This highlights the critical role of JAK signaling.

LIF also acts directly on oocyte nuclear and cytoplasmic maturation through the MAPK3/1 by regulating microRNA-21 (miR-21), thereby supporting early embryonic development [15,109]. STAT3 inhibitor induced a reduction in primary miR-21 (pri-miR-21) expression, negatively affecting cumulus cell expansion in bovine [167] and in mice [168].

MAPK activation also occurs via recruitment of the protein tyrosine phosphatase 2 (SHP2) [169]. Inhibition of SHP2 with the selective inhibitor, PHPS1, significantly reduced the oocyte maturation rates and decreased the number of ICM cells in bovine blastocysts, highlighting its indispensable role in sustaining oocyte competence and developmental potential [170]. SHP2 also contributes to PI3K/AKT activation, essential for embryonic development [171]. PI3K, in turn, when activated by LIF via JAK1, activates AKT by stimulating mTOR, which is important for regulating the cell cycle and metabolism. This process is essential for maintaining stem cells in an undifferentiated state, preventing them from prematurely specializing into specific cell types [172,173].

IGF1 induces PI3K and MAPK pathways by phosphorylation of its receptor IGF1R, serving as an anchor for other signaling molecules such as the insulin receptor substrate (IRS) [142] (Figure 1C). The use of a PI3K inhibitor, LY294002, after the addition of 100 ng/mL IGF1 in follicular tissue culture, inhibited the activation and growth of primordial follicles in sheep, also reducing AKT phosphorylation [147]. Stimulation of the PI3K/AKT/mTOR pathway by IGF1 increases mitochondrial activity in oocytes, promotes cell proliferation, reduces DNA damage, and supports the energy and nutrient supply required for embryonic development [142,155].

AKT and mTOR proteins are also essential for microtubule assembly and meiotic spindle formation, in addition to being critical for embryo development [171,174]. IGF1 promotes cumulus cell expansion in response to FSH through the PI3K/AKT and MAPK3/1 pathways, improving oocyte quality and consequently enhancing embryonic development and blastocyst formation [162,171]. In pigs, the PI3K/AKT pathway participates in regulating intracellular communication through connexin 43 phosphorylation and FSH stimulation [162]. The expression of *AKT1* was significantly higher in pig oocyte supplemented with FLI [175], promoting cumulus cell expansion by controlling the expression of hyaluronan synthase 2.

IGF1 can recruit the Src homology 2 (SH2) domain of the adaptor protein growth factor receptor-bound protein 2 (GRB2), thereby activating Ras and MAPK phosphorylation [140], facilitating meiosis resumption via gonadotropins and the transition from metaphase I to metaphase II in bovine oocytes [174].

In pig oocytes, FLI supplementation elevated MAPK levels at 22 h but not at 42 h of maturation, and inhibition with PD0325901 blocked meiotic progression [4]. MAPK accumulation required gonadotropins, as FLI alone was insufficient, though elevated MAPK was still observed in FLI-treated oocytes under certain conditions [21]. Another study showed that MAPK3/1 phosphorylation occurred within the first hour of pig oocyte maturation with FLI supplementation, while elevated AKT levels were maintained up to 16 h [171]. Although early development was similar across groups, from 24 h onward, FLI accelerated maturation via MAPK3/1 and AKT signaling.

Similarly, in mice, the levels of PI3K/AKT, MAPK3/1, and STAT3 were higher after 6 h of in vitro maturation following FLI treatment [176]. Genes related to EGF-like factors, such as ereg and areg, also showed increased expression in response to FLI. EGF-like growth factors regulate maternal mRNA translation via MAPK3/1 and can enhance zygote competence through activation of the PI3K/AKT pathway [177]. FGF2, LIF, and IGF1 activate multiple signaling pathways in a cross-linked manner, making their combined use highly effective for oocyte maturation and embryo culture. These benefits are especially clear in demanding applications such as nuclear transfer, gene editing, and vitrification. In chemically defined systems, these effects are amplified, and combining FLI with other additives that act on complementary pathways offers a promising strategy to further improve developmental outcomes.

## 4. The Use of FLI Medium in Different Species

### 4.1. Pig

The first study involving FLI supplementation in IVM was conducted in pigs [4]. These cytokines and growth factors are naturally present in follicular fluid, a common supplement in IVM [178,179,180]. Follicular fluid is a natural source of hormones, nutrients, and cytokines, but its use presents some limitations [181]. The incomplete characterization of its composition, combined with batch-to-batch variability, underscores the need for chemically defined media with controlled and reproducible formulations [181,182]. In addition, follicular fluid may also contain meiotic inhibitors, such as hypoxanthine, which can disrupt nuclear and cytoplasmic maturation, resulting in incomplete oocyte development and low-quality embryos [181,182,183].

**Figure 2:**
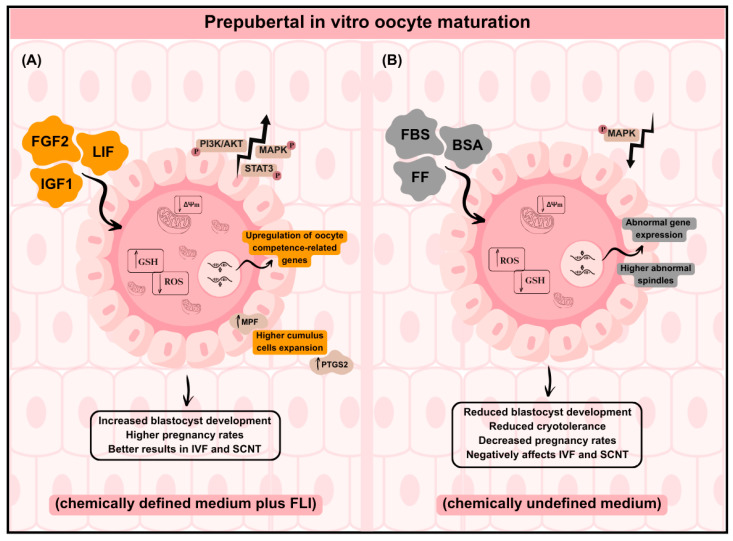
Effects of FLI supplementation during prepubertal in vitro oocyte maturation. (**A**) Supplementation with a chemically defined IVM medium containing FLI promotes the activation of key signaling pathways and enhances metabolic and gene expression-related factors, leading to improved embryo development. (**B**) The use of a chemically undefined IVM medium is associated with impaired signaling activation, redox imbalance, and abnormal gene expression, resulting in reduced in vitro embryo developmental outcomes. Solid black arrows indicate direct actions of supplements on oocytes and cumulus cells. Upward and downward curved black arrows represent upregulation and downregulation of signaling pathways, respectively. Upward and downward solid black arrows within the boxes indicate increased and decreased levels of glutathione (GSH), reactive oxygen species (ROS), and/or mitochondrial membrane potential (ΔΨm), respectively. Upward arrows within the brown shapes and nuclei indicate transcriptional regulation. This schematic model integrates data reported in bovine and porcine systems [4,186,188]. Schematic illustration created using Canva software 2025 (Canva Pty Ltd., Sydney, Australia).

The beneficial effects of FLI supplementation in prepubertal oocytes have been reported in several pig studies [2,4,21,175], particularly when compared with prepubertal oocytes cultured in chemically undefined IVM media [184,185,186]. Prepubertal oocytes offer a promising strategy for exploiting animals of high genetic value before sexual maturity [187]; however, their immature follicular environment compromises cytoplasmic maturation in vitro. Numerus defects including altered organelle distribution, redox imbalance, abnormal gene expression, reduced MAPK signaling, and metabolic dysregulation have been widely described in different species, such as pigs and cattle, leading to lower blastocyst production, reduced cryotolerance, and decreased pregnancy outcomes [184,185,186,188,189] (Figure 2).

FGF2, LIF, and IGF1 individually increased prepubertal pig oocyte maturation but at optimized concentrations (40, 20, and 20 ng/mL, respectively), these factors combined significantly enhanced both maturation and blastocyst production [4] (Table 1). The more consistent improvements observed in porcine systems, affecting both blastocyst yield and embryo quality, likely reflect the optimization of FLI concentrations in pigs. When extrapolated to other species, the same formulation may not achieve maximal quantitative responses. Nevertheless, the recurrent modulation of gene expression and quality-related markers across species suggest that the biological activity of FLI is preserved, even when increases in embryo number are not consistently observed. Similarly, the individual supplementation of FGF2 or IGF1 in pig oocytes increased MII percentages to 91%, while LIF alone raised the rate to 95%. Remarkably, the FLI medium yielded a maturation rate of 97%, demonstrating the synergistic effect of these components [171].

Follicle size affects developmental potential, with larger follicles generally showing higher competence. However, FLI supplementation improved outcomes in both large and small follicles, markedly enhancing maturation (86% vs. 77%), cleavage (80% vs. 77%), and blastocyst rates (31% vs. 29%) [175]. Small follicles supplemented with FLI also showed a significantly higher maturation (77.7% vs. 49%), cleavage (77% vs. 44%), and blastocysts (29% vs. 10%) compared with no FLI supplementation.

Studies consistently show that FLI supplementation enhances oocyte maturation and embryo development. Oocyte maturation increased to 89% with FLI compared to 55% in the controls, and the blastocyst rates reached 49.7% versus 38% [4]. FLI also improved the maturation rates (49% vs. 13% with DMEM) and achieved a pregnancy rate of 87% [190]. More recent studies confirmed these benefits even in BSA containing media, with the maturation (92% vs. 83%), cleavage (90% vs. 78%), and blastocyst (45% vs. 32%) rates significantly improved [2]. High maturation (73%), cleavage (78%), and blastocyst formation (33%) were also reported [191], while FLI outperformed porcine follicular fluid for maturation (70% vs. 61%) and blastocyst production (15.7% vs. 14.3%) [179]. While FF contains FGF2, LIF, and IGF1 in its composition, these factors do not act synergistically in the in vitro environment, likely due to variations in the composition of follicular fluid [179].

The combination of gonadotropins (GN), FSH and LH, with FLI significantly increased the maturation rates to over 80%, which is reflected in improved cleavage rates (over 30%) and blastocyst rates (around 30%) [21]. This further supports the positive impact of FLI and its synergistic potential with FSH and LH. Notably, FSH and LH are crucial for initiating the early stages of cumulus cell expansion, and in the presence of FLI, both promote substantial cumulus cell expansion around 42 h of maturation. Interestingly, COCs matured without GN but in the presence of FLI showed limited cumulus cell expansion, yet oocyte maturation and blastocyst development were unaffected. These results highlight that FLI plays a critical role in supporting oocyte competence while also amplifying cumulus cell expansion when GN is present, exerting a direct effect on the oocyte itself and not only through the cumulus cells.

**Table 1:** Effect of FLI supplementation (FGF2 + LIF + IGF1) on in vitro maturation of prepubertal pig oocytes and subsequent embryo development. Summary of IVM media composition and developmental outcomes reported in studies that used only FLI as a supplement. Values represent the mean percentages of MII oocytes, cleaved embryos, and blastocyst formation reported in each publication. Unless otherwise specified, all studies used 40 ng/mL FGF2 + 20 ng/mL LIF + 20 ng/mL IGF1. Different letters in the maturation, cleavage, and blastocyst results indicate statistically significant differences (*p* < 0.05).

Reference	Medium	Groups	Maturation	Cleavage	Blastocyst
Yuan et al., 2017 [4]	TCM199-based medium (serum-free) supplemented with 10 ng/mL EGF, 0.57 mM cysteine, 50 µg/mL gentamicin, and ± FLI	Control	55% (a)	n.i	38% (a)
		FLI	89% (b)		49.7% (b)
Serrano Albal et al., 2022 [192]	POM supplemented with FSH (0.5 IU/mL), LH (0.5 IU/mL) and dbc-AMP (0.1 mM) for 20 h. COCs were cultured in the same medium but without hormones and dbc-AMP for a further 24 h	Control	62.3% (a)	n.i	10.8% (a)
		FLI	70.9% (a)		15.7% (a)
		sFF (10%)	61.5% (a)		14.3% (a)
		sFF + FLI	60.3% (a)		12.0% (a)
Redel et al., 2021 [21]	TCM199 containing 3.05-mM glucose, 0.91-mM sodium pyruvate, 0.57-mM cysteine, 10-ng/mL EGF, 10-μg/mL gentamicin, 0.1% polyvinyl alcohol ± 0.5-μg/mL LH and 0.5-μg/mL FSH, and ±FLI	−GN −FLI	50% (c)	15% (a)	10% (b)
		−GN +FLI	78% (a)	29% (a)	25% (a)
		+GN −FLI	66% (b)	20% (a)	25% (a)
		+GN +FLI	82% (a)	32% (a)	30% (a)
Procházka et al., 2021 [171]	TCM199 with 0.2 mM sodium pyruvate, 6.85 mM L-glutamine, 0.57 mM cysteine, 50 μg/mL gentamycin, 1 mg/mL BSA, 10 IU/mL PMSG, 10 IU/mL hCG, 10 ng/mL EGF, ±40 ng/mL FGF2, 20 ng/mL IGF1 and 2 μL/mL LIF	Control	68.05% (a)	70.51% (a)	20.69% (a)
		FLI	95.38% (b)	87.88% (b)	34.07% (b)
Murin et al., 2023 [183]	TCM199 with 0.005% gentamicin, 0.0022% sodium pyruvate, 0.01% L-glutamine, 0.1% BSA, 10 ng/mL EGF, 40 ng/mL FGF2, 20 ng/mL IGF1, 2000 IU/mL LIF, 0.57 mM L-Cysteine, 10 IU/mL PMSG and 10 IU/mL hCG	FLI	73%	78%	33%
Rosenbaum Bartkova et al., 2024a [2]	DMEM—with 50 ng/mL EGF, 10 IU/mL PMSG + hCG; FLI—TCM199 with 10 ng/mL EGF, FLI, 10 IU/mL PMSG + hCG, plus cysteine, gentamicin, BSA, and pyruvate.	DMEM	83.6% (a)	78% (a)	32.5% (a)
		FLI	92.4% (b)	90.2% (b)	45.7% (b)
Bartková et al., 2024b [175]	TCM199 with 0.2 mM sodium pyruvate, 6.85 mM L-glutamine, 0.57 mM cysteine, 50 μg/mL gentamycin, 1 mg/mL BSA, 10 IU/mL PMSG, 10 IU/mL hCG, 10 ng/mL EGF, ±40 ng/mL FGF2, 20 ng/mL IGF1 and 2 μL/mL LIF	SF (Control)	49.6% (b)	44.4% (b)	10.9% (b)
		LF (Control)	79.7% (a)	71.6% (a)	23.6% (a)
		SF (FLI)	77.7% (a)	77.3% (a)	29.2% (c)
		LF (FLI)	86.9% (a)	80.0% (a)	31.6% (c)

POM: porcine oocyte medium; sFF: swine follicular fluid; n.i: not informed; GN: gonadotrophin; SF: small follicle; LF: large follicle. Letters a, b, and c: indicate statistical significance (*p* < 0.05). (+): presence of GN or FLI; (−): absence of GN or FLI; (±): medium with or without FLI.

The transcript profile modulated by FLI supplementation demonstrates the significant role in supporting nuclear and cytoplasmic maturation in pig oocytes [191]. Among these genes, *ANTRX1* (cell adhesion), *FLNA* (filamin A), and *TPM3* (tropomyosin 3) are involved in the reorganization of the actin cytoskeleton. Specifically, FLNA and TPM3 play a crucial role in forming the meiotic spindle in mouse oocytes [191,193], with FLNA being responsible for actin filament polymerization during meiotic maturation. Among the most highly expressed genes across all four stages of oocyte development, the DNA methyltransferase I and SIN3 transcription regulator family member A genes stand out. These are associated with DNA methylation and cell cycle regulation, respectively, highlighting their pivotal roles in ensuring proper oocyte maturation and developmental competence [191].

FLI supplementation during maturation also influences the transcript profile observed in embryos. A proteomic profile of oocytes cultured in DMEM or FLI and their subsequent embryos identified important biological pathways in both groups, such as gene expression regulation (DNA replication, transcription, and translation), cytoskeleton organization, microtubules and meiotic spindle formation, and zona pellucida [2]. It is known that DMEM already contains FGF2 and IGF1 at concentrations of 5 ng/mL and 100 ng/mL, respectively, but does not contain LIF, which differentiates it from the FLI medium formulation [4]. The 4-cell stage embryos treated with FLI presented transcripts that, in DMEM, were only observed at the 8-cell stage [2]. This clearly shows that FLI not only favors early embryonic development but also optimizes the in vitro environment by providing enhanced molecular support through the early activation of genes crucial for embryonic progression. This results in the production of blastocysts with greater efficiency and superior quality.

The transcriptional profiles in oocytes during the different stages of oocyte maturation supplemented with FLI further reinforces the individual and synergistic action of the components in this cocktail. This molecular signature highlights the comprehensive influence of FLI, not only promoting nuclear and cytoplasmic maturation but also creating an environment conducive to optimizing oocyte competence, reflecting the harmonious interaction of the embryotrophic factors present.

### 4.2. Cattle

A few studies have explored the effects of FLI supplementation in bovine in vitro environments, highlighting its influence on both oocyte maturation and embryo culture. During adult oocyte IVM, FLI supplementation impacted TZPs over time and increased the number of mature oocytes, also producing a higher number of blastocysts with reduced lipid content (*p* < 0.05), an essential factor for successful cryopreservation [194]. This was further supported by higher survival rates post-thawing, increased hatching of embryos (19.8% in the control group vs. 40.4% in FLI-treated embryos, *p* < 0.05), and a lower number of apoptotic cells.

Embryo cryopreservation techniques still yield pregnancy rates up to 10% lower than those achieved with non-frozen embryos, limiting the effectiveness of this approach [194]. To address this challenge, FLI supplementation has been studied to assess its impact on embryos cryopreserved through slow-rate freezing [191,195,196]. Stoecklein et al. [194] evaluated the effect of FLI supplementation during embryo culture of post-cryopreserved embryos and observed an increased number of embryos developing into blastocysts following FLI treatment. However, no significant effects were observed on pregnancy outcomes or developmental competence, nor was there any statistical difference in the number of embryos recovered 15 days after implantation.

Recently, McDonald et al. [196] demonstrated the positive effects of FLI supplementation on bovine embryo transfer outcomes. Embryos cultured with FLI did not show statistically significant differences in blastocyst rates (48.73% vs. 34.92%) or pregnancy rates (37.9% vs. 30.9%) compared to the controls. However, on Day 24 of pregnancy, circulating pregnancy-associated glycoprotein levels, an important marker of pregnancy and placental function, were significantly higher in recipient cows that received FLI-treated embryos (*p* < 0.05) [196]. This suggests that FLI may support placental development and enhance embryo viability beyond day 30 of pregnancy.

The embryos cultured in FLI also showed the upregulation of genes such as *PTGS2*, nitric oxide synthase 2, and toll-like receptor 2, along with other genes associated with interferon signaling, cellular responses to stress, and inflammation [196]. In cattle, between days 12 to 15, the embryo undergoes the elongation phase with high secretion of interferon tau (IFNT). Between days 8 to 28, there is a loss of up to 20% of embryos produced in vitro due to the critical elongation phase and associated hormonal changes [87,197]. The presence of PTGS2 and interferon are important indicators for maternal recognition of pregnancy and subsequent embryo survival after implantation. Thus, the upregulation of these genes in the FLI-treated embryos suggests a higher likelihood of embryo survival following cryopreservation.

Although no differences in embryonic yield were observed between the FLI-treated and control embryos [196], the expression profile associated with FLI treatment indicates potential for improved conceptus development and pregnancy signaling. This distinction reinforces the need to evaluate embryo quality beyond numerical yield, particularly in applications such as embryo transfer or genome editing.

The use of FLI medium in cattle also enables cells to be used for more advanced reproductive techniques, such as SCNT [15]. In SCNT embryos, an increase in the number of trophectoderm and ICM cells was observed after FLI treatment, leading to a higher pregnancy rate. Among the 30 SCNT embryos treated with FLI, seven full-term cloned calves were produced, none exhibiting clinical characteristics of LOS. In contrast, one animal in the control group presented LOS, indicating that FLI in the maturation medium improved both the pregnancy rates and full-term development, resulting in a greater number of healthy cloned animals.

The positive effects of FLI were also observed when added to bovine oocyte maturation medium, where maturation levels reached 80% compared to 66.5% in the untreated medium, along with reduced reactive oxygen species [15]. Furthermore, the impact of FLI components on nuclear and cytoplasmic maturation of bovine oocytes was recently demonstrated [164]. Oocytes supplemented with FLI exhibited higher maturation rates, indicated by a decreased number of germinal vesicles (GV) and an increased number of cortical granules, suggesting that these embryotrophic factors improved oocyte quality for in vitro production.

Studies conducted on bovine models have shown variability in the extent to which FLI supplementation improves blastocyst yield, although enhancements in oocyte and embryo quality are more consistently reported. Such discrepancies may partially reflect the lack of species-specific optimization of FGF2, LIF, and IGF1 concentrations for the bovine in vitro system. For instance, FGF2 mRNA expression in bovine ovarian follicles varies according to follicular size, and follicular fluid concentrations have been reported as low as 0.62 ng/mL in Bali cattle [72]. This marked difference between physiological levels and the concentration commonly used in FLI supplementation (40 ng/mL FGF2) may contribute to variability in developmental outcomes observed across studies.

### 4.3. Other Species

Studies applying the FLI cocktail in other species are still scarce but hold great potential for improving the efficiency of in vitro embryo production in species with inherently low developmental rates, such as buffalo. The blastocyst rate in buffalo is approximately 10–20%, significantly lower than the 30–40% observed in cattle [198,199]. This reduced developmental competence poses challenges to the use of buffalo in conservation and assisted reproduction studies. Mesenchymal stem cells present a promising alternative, as they secrete various growth factors and cytokines, including FGF2, LIF, IGF1, EGF, VEGF, and others [200]. The conditioned medium derived from these stem cells can create an enriched and supportive environment for the in vitro development of buffalo embryos, thereby enhancing the efficiency of the process.

A study conducted prior to Yuan et al. [4], in buffalo, demonstrated the potential of a conditioned medium obtained from the culture of umbilical cord embryonic cells for in vitro embryo culture. This medium, enriched with FLI, resulted in significant improvements in embryonic development rates [199]. The in vitro culture medium supplemented with 50% conditioned medium increased the cleavage rates to 70% and blastocyst rates to 24%. These findings underscore the critical role of these factors in embryonic development and highlight the potential of conditioned medium as a tool to address the limitations in the in vitro production of buffalo embryos.

Species such as equine and canine are characterized by higher embryonic loss and low IVM efficiency, respectively. A preliminary study in equine indicated that FLI-treated oocytes tended to improve the cleavage rates compared with the controls and numerically increased the number of pregnant mares [201]. Also, insulin-transferrin-selenium (ITS) supplementation during canine IVM increased nuclear maturation by 20%, improved membrane integrity, and reduced ROS levels [202]. Similarly, ITS, FLI, and other additives enhanced nuclear maturation in prepubertal porcine oocytes [203]. These findings raise the possibility that the limited efficiency observed in these species may be partially linked to suboptimal support of key signaling and metabolic pathways during IVM. The absence of FLI-based investigations therefore represents a relevant gap in the field and highlights the need for mechanistically guided, species-specific optimization strategies, particularly in IVP systems that remain technically challenging.

In mice, the individual supplementation of FGF2, LIF, or IGF1 in the maturation medium did not significantly improve embryo quality [176]. However, consistent with findings in other species, the combination of all three factors was more effective, resulting in a higher number of hatched blastocysts and an improved embryo quality. This improvement was further evidenced by a greater number of implanted embryos following transfer and an increased number of fetuses developed per recipient.

Based on these results, Amargant et al. [204] performed IVM with juvenile human COCs and denuded oocytes, obtained from ovarian tissue preservation, in a medium containing FLI. Treatment with FLI showed a slight improvement in MII rates after in vitro maturation in COCs, which was not observed in denuded oocytes. Similar to the findings of Yuan et al. [4], a significant progressive increase in cumulus cell expansion was observed from the beginning of maturation until 16 h, which was reflected in the number of TZP retractions per oocyte and in MAPK protein levels.

The limited number of human studies likely reflects the ethical and regulatory constraints inherent to clinical reproductive research, which substantially restrict experimental manipulation compared to animal models. Unlike livestock systems, where supplementation strategies can be systematically tested under controlled experimental conditions, human assisted reproduction technology operates within strict clinical and ethical boundaries that limit dose exploration, combinatorial testing, and invasive molecular assessments. As a result, translational advances from animal models to human systems often remain indirect, and the evaluation of novel supplementation strategies in humans must balance biological potential with clinical safety and regulatory oversight. This potential is particularly relevant in the context of ovarian tissue cryopreservation, a strategy used for children with cancer, as treatment with radiation and chemotherapy can induce ovarian failure or even early menopause [205].

## 5. Association of FLI Medium with Other Additives Improves Embryo Quality

The addition of supplements such as amino acids, cytokines, and antioxidants has been widely used to enhance outcomes of in vitro embryo production [14,206,207]. Some of these additives are naturally present in the ovary, follicular fluid, and granulosa cells of antral follicles [31,208,209]. For example, C-type natriuretic peptide (CNP) and melatonin play crucial roles, promoting the resumption of meiosis and reducing intracellular oxidative stress by decreasing ROS levels, respectively [10,210]. In synergy with IGF1, melatonin can increase GSH levels in bovine oocytes, boosting the antioxidant defense system [9].

In cattle, the addition of CNP and melatonin to FLI medium demonstrated positive results by influencing cAMP levels for up to 8 h, promoting the resumption of oocyte meiosis and improving the number of TZPs [10]. Genes associated with oxidative stress showed a significant reduction after the in vitro treatment of oocytes with FLI, CNP, and melatonin [10]. Furthermore, increased expression of genes related to cumulus cell expansion was observed, along with genes associated with mitochondrial function and anti-apoptosis, which were more highly expressed in blastocysts obtained with FLI-CNP-melatonin supplementation compared to the control group.

An IVM medium supplemented with FLI, citrate, porcine follicular fluid (pFF), and ITS (referred to as FIpc) showed a significant improvement in polar body extrusion of prepubertal porcine oocytes (86.6%) compared with FLI supplementation alone (68.4%) (*p* < 0.05) [203]. Similar results were observed in small follicles (<3 mm) (69.8% FIpc vs. 60.7% FLI, *p* < 0.05) and medium follicles (3–6 mm) (88.1% FIpc vs. 74.5% FLI, *p* < 0.05). This cocktail also upregulated the expression of cumulus expansion and oocyte maturation markers, and showed improved cortical granule distribution, proper chromosomal alignment, and higher levels of GSH.

ITS supplementation has been shown to promote glucose uptake through insulin while reducing oxidative damage via transferrin and selenium, leading to improved oocyte and embryo quality [14,203]. However, in prepubertal lamb oocytes, the use of FLI in combination with ITS did not further improve the blastocyst rates (43% FLI vs. 42% FLI + ITS). A similar trend was observed with sericin supplementation, where FLI alone achieved comparable results to FLI combined with sericin [30].

Similarly, Currin et al. [31] observed in prepubertal porcine oocytes that the addition of a cocktail containing ITS, melatonin, and cysteine to FLI medium and follicular fluid did not result in significant improvements in oocyte and embryo development. However, when different combinations of these additives were associated with FLI, a trend toward improvement in cumulus cell expansion, cleavage, and blastocyst rates was observed. The inclusion of 20% follicular fluid during IVM, along with FLI supplementation, increased the rates of MII, blastocyst formation, and hatched blastocysts. These findings suggest that the beneficial effects of FLI may become more evident in prepubertal oocytes, which typically exhibit lower intrinsic developmental competence and may therefore rely more strongly on optimized culture conditions and signaling support during maturation (Figure 2).

Despite the critical role of these additives in the in vitro environment, their supplementation to FLI medium has yielded mixed results. This inconsistency may be attributed to compensation between the factors present, the need for optimized concentrations of these additives, or the specific activation or inhibition of molecular pathways when these components are combined. Nevertheless, even though current studies have not consistently demonstrated significant improvements from the combination of additives with FLI medium, integrating FLI with antioxidants, amino acids, or other supplements remains a promising approach for enhancing oocyte maturation processes across different species, aiming to improve the quality of embryos produced in vitro.

## 6. Conclusions

In summary, active research on the use of FLI supplementation emphasizes its potential impact on enhancing embryo production. Existing studies consistently demonstrate its benefits for both oocyte maturation and embryo quality across various species, especially its capacity on improving prepubertal oocyte maturation. These findings are supported by the upregulation of transcriptomic and proteomic profiles, as well as the activation of key signaling pathways. The potential of FLI extends beyond improving in vitro embryo production, as it represents a promising approach for routine use in nuclear transfer and gene-editing procedures, which often show lower success rates compared to conventional in vitro embryo production.

However, the variability in concentrations and composition of medium used with individual FGF2, LIF, and IGF1 supplementation likely represents a major source of the inconsistent outcomes reported within and across species. Although the FLI formulation (40 ng/mL FGF2, 20 ng/mL LIF, and 20 ng/mL IGF1) was originally optimized in porcine models, it has been broadly adopted in bovine, murine, lamb, and human systems without species-specific recalibration. This cross-species extrapolation assumes conserved physiological requirements that may not reflect the actual concentrations present in the follicular fluid or reproductive tract of each species. Consequently, the standardized use of FLI across species may limit its biological efficacy and contribute to variability in developmental outcomes.

To fully harness the potential of FLI, future research must prioritize the determination of species-specific physiological concentrations of these growth factors and cytokines. Such precision-based optimization is essential to establish reproducible, biologically relevant, and consistently effective supplementation strategies across experimental systems. Looking ahead, the combination of FLI with other additives holds great promise for further enhancing these outcomes, paving the way for broader applications and increased efficiency in reproductive biotechnologies.

## Data Availability

All data discussed in this review are from published studies and are available from the original sources cited in the manuscript.
